# Optimization and evaluation of surface-enhanced laser-desorption/ionization time-of-flight mass spectrometry for protein profiling of cerebrospinal fluid

**DOI:** 10.1186/1477-5956-4-7

**Published:** 2006-04-27

**Authors:** Nelson Guerreiro, Baltazar Gomez-Mancilla, Stéphane Charmont

**Affiliations:** 1 Novartis, BioMarker Development, Exploratory Development, Klybeckstasse, CH-4002 Basel, Switzerland

## Abstract

Cerebrospinal fluid (CSF) potentially carries an archive of peptides and small proteins relevant to pathological processes in the central nervous system (CNS) and surrounding brain tissue. Proteomics is especially well suited for the discovery of biomarkers of diagnostic potential in CSF for early diagnosis and discrimination of several neurodegenerative diseases. ProteinChip surface-enhanced laser-desorption/ionization time-of-flight mass spectrometry (SELDI-TOF-MS) is one such approach which offers a unique platform for high throughput profiling of peptides and small proteins in CSF. In this study, we evaluated methodologies for the retention of CSF proteins < 20 kDa in size, and identify a strategy for screening small proteins and peptides in CSF. ProteinChip array types, along with sample and binding buffer conditions, and matrices were investigated. By coupling the processing of arrays to a liquid handler reproducible and reliable profiles, with mean peak coefficients of variation < 20%, were achieved for intra- and inter-assays under selected conditions. Based on peak *m/z *we found a high degree of overlap between the tested array surfaces. The combination of CM10 and IMAC30 arrays was sufficient to represent between 80–90% of all assigned peaks when using either sinapinic acid or α-Cyano-4-hydroxycinnamic acid as the energy absorbing matrices. Moreover, arrays processed with SPA consistently showed better peak resolution and higher peak number across all surfaces within the measured mass range. We intend to use CM10 and IMAC30 arrays prepared in sinapinic acid as a fast and cost-effective approach to drive decisions on sample selection prior to more in-depth discovery of diagnostic biomarkers in CSF using alternative but complementary proteomic strategies.

## Background

Human cerebrospinal fluid (CSF) is largely produced by the highly vascular choroid plexus [1]. CSF continuously circulates through cavities in the brain and spinal chord and in the subarachnoid space, and contains peptides and proteins that play critical roles in many physiological processes [2]. Its proximity to the brain and the little risk involved with procuring CSF samples from individuals makes it an appropriate source of protein biomarkers for neurodegenerative disease. CSF is in direct contact with the extracellular space of the brain, and so contains some proteins and other products of neural cell origin. As such, any variation in protein composition or abundance relative to normal CSF may potentially reflect pathological processes in the surrounding brain tissue and other parts of the central nervous system (CNS) [1,3-6]. Monitoring a combination of biomarkers in CSF exhibiting high sensitivity and specificity offers the potential for aiding early stage diagnosis, when correct diagnosis is often difficult, and when therapeutic compounds have the greatest potential for being effective. Biomarkers could also be used as quantitative indices of disease progression and response to therapeutics, and for discriminating early or incipient Alzheimer's disease (AD) from age-associated memory loss impairment, depression, and some secondary dementias.

Similar to plasma, the predominant proteins in CSF are isoforms of serum albumin, transferin and immunoglobulins, which represent more than 70% of the total protein amount. Furthermore, an unwanted high dynamic range of protein abundance is found in CSF, making the detection of lower abundance proteins extremely challenging with the current analytical methods. An additional challenge with analyzing CSF is protein concentration. On average CSF contains 100 fold less protein than plasma, therefore, necessitating the need for larger sample amounts relative to plasma. A variety of proteomic approaches have recently been used to characterize the peptide and protein composition in CSF. Most of these proteomic technology platforms are centered around the implementation of mass spectrometric techniques in conjunction with several other analytical techniques such as gel electrophoresis, isoelectric focusing, and liquid chromatography (LC) [3,7-11]. While these approaches provide a large amount of data and can identify hundreds of proteins, they are generally very time consuming and hence restrictive in the number of comparative samples that can be analyzed.

Surface-based enrichment approaches in combination with MS is one such approach which offers a unique platform for high throughput CSF protein profiling. ProteinChip surface enhanced laser desorption/ionization time-of-flight mass spectrometry (SELDI-TOF-MS) technology (Ciphergen Biosystems Inc., Fremont, CA) was developed to facilitate the high-throughput analysis of proteins in complex biological samples such as body fluids [12-17]. This technology uses chip-based protein sample arrays with different chromatographic surfaces designed to capture and retain subsets of proteins based on specific protein characteristics such as affinity, charge, hydrophobicity, and metal-binding capabilities. After a series of binding and washing steps of the chromatographic surfaces, matrix is added to the spots and the samples are analyzed by laser desorption/ionization-TOF-MS generating mass/charge profiles of the applied sample [18]. Proteomic expression patterns derived from mass spectrometry have been put forward as potential biomarkers of clinical relevance [19-21]. Such spectral profiles can be compared to uncover patterns of differential abundance and aid in the identification of diagnostic patterns of disease and toxicity [13,15,22-25]. Moreover, surface-based enrichment approaches have the potential to capture and enrich for low abundant, low molecular weight species [12,17,22,26,27]. The low molecular weight region of CSF comprising of peptides and fragments of proteins remains relatively unexplored and represents a potential treasure trove of histopathological information.

Although the ProteinChip SELDI-TOF approach is a straightforward, robust platform for high throughput protein profiling, much has been discussed concerning its poor representation of the proteome, particularly for proteins above 20 kDa in mass. In this paper, however, we take advantage of the technologies potential for screening peptides and small proteins between the 2–20 kDa mass window range, and its requirement of only small sample volumes for analysis. As with any technology, experimental procedures must be optimized and reproducible to ensure consistent data output. Therefore, the aim of this paper was to improve on existing methodologies to identify effective conditions of retention for profiling proteins in the low molecular weight region of the CSF proteome.

## Results

### Assessment of ProteinChip array types, buffer conditions and matrix for profiling CSF

**Figure 1:**
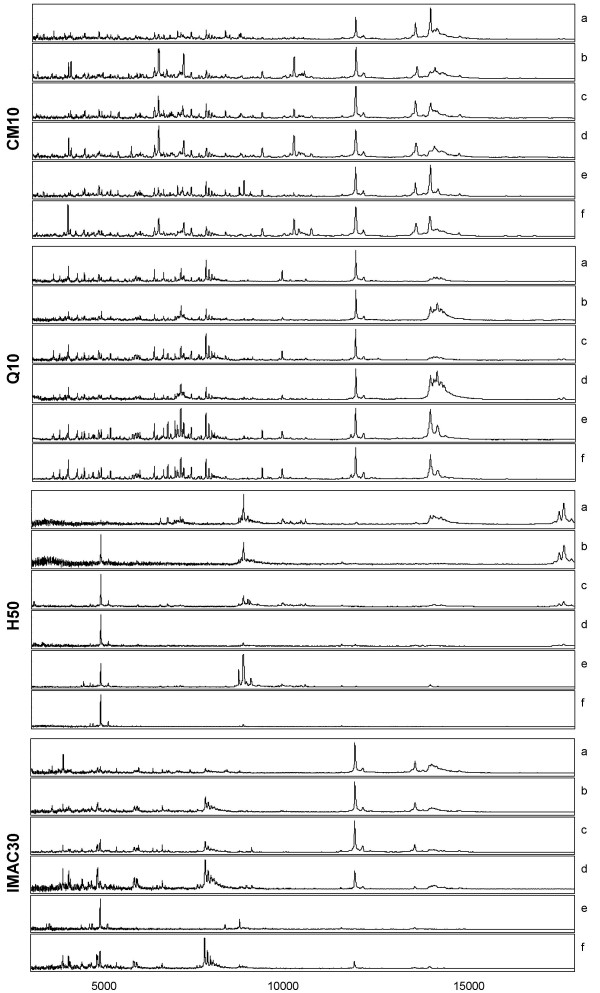
**Representative SELDI-TOF MS spectra of pooled CSF sample obtained from all tested conditions using SPA as matrix. **CSF was processed on CM10, Q10, H50 and IMAC30 ProteinChip arrays prepared under the following conditions: **CM10 **a: Native – 100 mM ammonium acetate, pH 4.0 b: Native – 100 mM ammonium acetate/0.1% Triton X-100, pH 4.0 c: Denatured – 100 mM ammonium acetate, pH 4.0 d: Denatured – 100 mM ammonium acetate/0.1% Triton X-100, pH 4.0 e: Denatured/reduced – 100 mM ammonium acetate, pH 4.0 f: Denatured/reduced – 100 mM ammonium acetate/0.1% Triton X-100, pH 4.0 **Q10 **a: Native – 100 mM Tris-HCl, pH 9.0 b: Native – 100 mM Tris-HCl/0.1% Triton X-100, pH 9.0 c: Denatured – 100 mM Tris-HCl, pH 9.0 d: Denatured – 100 mM Tris-HCl/0.1% Triton X-100, pH 9.0 e: Denatured/reduced – 100 mM Tris-HCl, pH 9.0 f: Denatured/reduced – 100 mM Tris-HCl/0.1% Triton X-100, pH 9.0 **H50 **a: Native – 10% AcN/0.1% TFA b: Native – PBS c: Denatured – 10% AcN/0.1% TFA d: Denatured – PBS e: Denatured/reduced – 10% AcN/0.1% TFA f: Denatured/reduced – PBS **IMAC30 **a: Native – 100 mM Cu sulfate,100 mM Na phosphate/0.5 M NaCl, pH 7.0 b: Native – 100 mM Ni sulfate, 100 mM Na phosphate/0.5 M NaCl, pH 7.0 c: Denatured – 100 mM Cu sulfate, 100 mM Na phosphate/0.5 M NaCl, pH 7.0 d: Denatured – 100 mM Ni sulfate, 100 mM Na phosphate/0.5 M NaCl, pH 7.0 e: Denatured/reduced – 100 mM Cu sulfate, 100 mM Na phosphate/0.5 M NaCl, pH 7.0 f: Denatured/reduced – 100 mM Ni sulfate, 100 mM Na phosphate/0.5 M NaCl, pH 7.0

**Figure 2:**
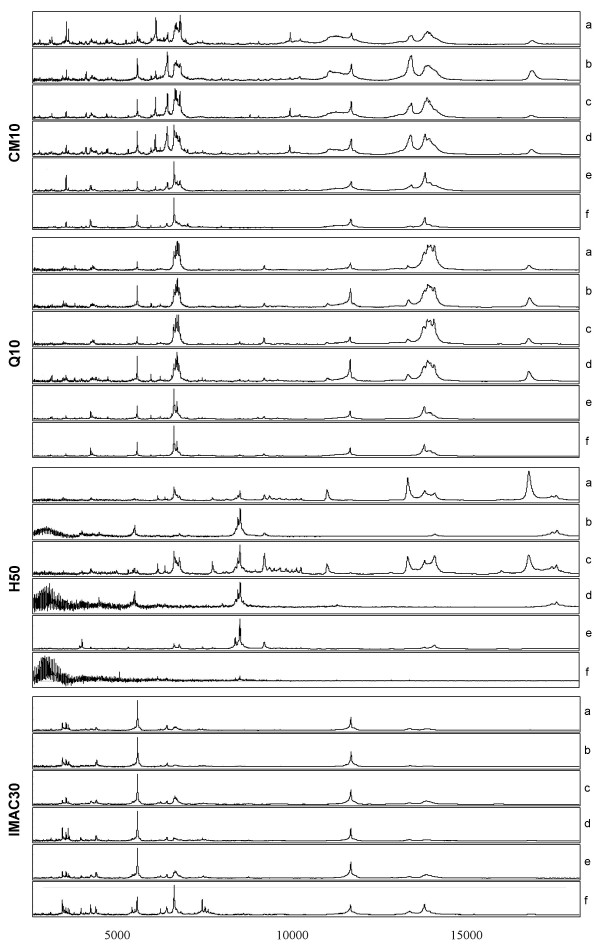
**Representative SELDI-TOF MS spectra of pooled CSF sample obtained from all tested conditions using CHCA as matrix. **CSF was processed on CM10, Q10, H50 and IMAC30 ProteinChip arrays prepared under the same conditions as described in figure 1, but with CHCA as the energy absorbing matrix.

Four ProteinChips (CM10, Q10, H50 and IMAC30) were used for the detection of proteins present in CSF between the *m/z *range of 2.5–20 kDa. In order to identify effective conditions of protein retention, pooled human CSF samples were prepared under native, denatured, and denatured/reduced conditions, and analyzed on ProteinChip arrays processed using different buffer conditions and matrices. For each condition of retention the samples were processed in triplicate. The selection criteria used to determine the choice of conditions for profiling CSF was dependent on both the number and quality of resolved peaks within the mass spectra. Tables 1 and 2 summarize the number of peaks automatically detected between the *m/z *range of 2.5–20 kDa across all tested conditions using either SPA or CHCA as the energy absorbing matrices. Peaks were required to have a signal to noise ratio of 3 or greater in order to be considered. The representative profiles obtained from the tested conditions on arrays processed with SPA and CHCA are shown in figures 1 and 2, for the respective matrices. Based on total peak count, denatured samples consistently demonstrated a higher number of resolved peaks when compared with samples prepared under native or denatured/reduced conditions. This was the case across all four ProteinChip arrays processed with either SPA or CHCA. When comparing peak count between CM10 and Q10 arrays, slightly better profiles were obtained with both array types processed in the absence of Triton X-100. In the case of IMAC30, surface activation with copper provided the higher peak number when compared to activation with nickel, whereas with H50, higher peak numbers were obtained with 10% AcN/0.1% TFA as binding buffer compared to PBS. Typically for the analysis of proteins and peptides by MS, SPA is the matrix of choice for large proteins, whereas CHCA is the preferred matrix for peptides (< 4 kDa). Not surprising, arrays processed with SPA consistently showed better peak resolution and higher peak number across all surfaces within the measured mass range of 2.5 to 20 kDa (tables 1 and 2).

**Table 1:** Comparison of peak count between all tested conditions using SPA as matrix.

		Native		9 M urea, 2% CHAPS		9 M urea, 2% CHAPS, 10 mM DTT	
		Peak count	Average	Peak count	Average	Peak count	Average

CM10	w/o Triton X-100	103	103	121	117	102	100
		104		112		90	
		101		119		99	
	Triton X-100	100	102	105	104	95	98
		104		105		102	
		101		102		90	

Q10	w/o Triton X-100	101	102	109	109	103	100
		103		108		97	
		102		110		100	
	Triton X-100	89	90	105	106	104	104
		90		105		104	
		91		108		103	

H50	10% AcN, 01% TFA	104	101	103	105	98	102
		99		103		109	
		100		109		101	
	PBS	76	79	74	72	77	76
		82		74		74	
		78		70		77	

IMAC30	Copper	102	99	100	113	97	103
		95		110		106	
		97		131		106	
	Nickel	107	105	88	92	84	81
		102		90		70	
		103		100		90	

Peaks with a signal-to-noise of 3 or greater were assigned between the *m/z *range of 2.5–20 kDa. 5 μL of pooled CSF prepared under native, denaturing, and denaturing/reducing buffer was applied in triplicate to each condition tested on CM10, Q10, H50, and IMAC30 arrays.

**Table 2:** Comparison of peak count between all tested conditions using CHCA as matrix.

		Native		9 M urea, 2% CHAPS		9 M urea, 2% CHAPS, 10 mM DTT	
		Peak count	Average	Peak count	Average	Peak count	Average

CM10	w/o Triton X-100	66	68	98	90	76	79
		70		86		85	
		69		86		78	
	Triton X-100	75	76	77	77	68	67
		76		84		68	
		75		72		66	

Q10	w/o Triton X-100	86	86	91	87	82	75
		86		89		74	
		86		82		70	
	Triton X-100	77	75	73	81	75	71
		73		89		70	
		74		81		70	

H50	10% AcN, 01% TFA	71	71	85	81	73	73
		72		80		73	
		71		80		73	
	PBS	60	62	63	63	59	58
		64		63		61	
		62		65		55	

IMAC30	Copper	93	89	99	104	77	82
		85		109		81	
		88		106		89	
	Nickel	90	89	87	87	74	81
		88		85		85	
		89		91		84	

Peaks with a signal-to-noise of 3 or greater were assigned between the *m/z *range of 2.5–20 kDa. 5 μL of pooled CSF prepared under native, denaturing, and denaturing/reducing buffer was applied in triplicate to each condition tested on CM10, Q10, H50, and IMAC30 arrays.

### Assessment of peak overlaps between ProteinChip array types

**Figure 3:**
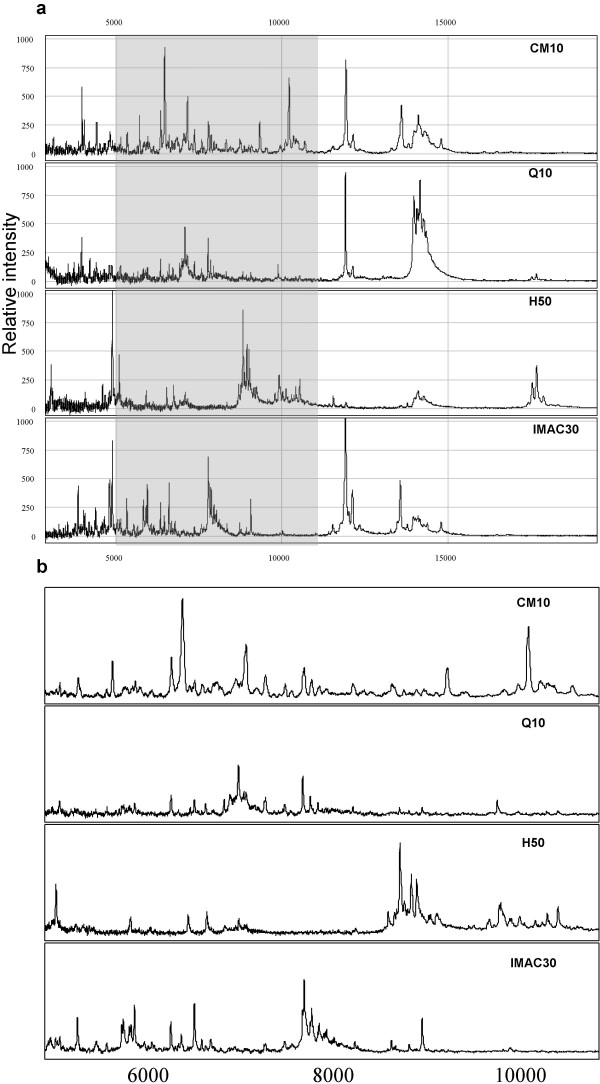
**Representative SELDI-TOF MS spectra of CSF in denaturing buffer on CM10, Q10, H50 and IMAC30 arrays. **a) CSF was diluted 1:1 in denaturing buffer 9.5 M urea, 2% CHAPS, 50 mM Tris-HCl, pH 9.0. Denatured CSF samples were diluted 1:4 in appropriate binding buffer and processed on: CM10 with 100 mM ammonium acetate pH 4.0; Q10 with 100 mM Tris-HCl pH 9.0; H50 with 10% AcN, 01% TFA; and IMAC30 with 100 mM Na phosphate, 0.5 M NaCl pH 7.0 and 100 mM Cu sulphate activation. ProteinChip arrays were prepared with SPA. b) Enlargement of the shaded spectra region showing peaks between *m/z *range of 4–11 kDa.

**Figure 4:**
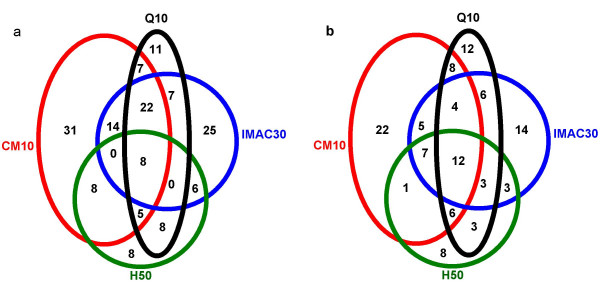
**Venn diagram representing the overlap of peaks between ProteinChip array types. **CSF prepared in denaturing buffer was processed on CM10, Q10, H50 and IMAC30 ProteinChip arrays using (a) SPA, and (b) CHCA. Peaks with a signal-to-noise ratio of 3 or greater, between the *m/z *range of 25–20 kD, were considered. When assigning peak clusters across spectra, two peaks on different surfaces were assumed to be the same protein if both their respective *m/z *were within 0.3%.

Peak profiles for CSF samples prepared in denaturing buffer were compared across all four ProteinChip array types to asses the extent of peak overlap between the different array types. The assessment of peak overlaps would be used to determine the optimal combination of array types, in terms of the number and resolution of peaks, to be adopted for a CSF profiling strategy. Figure 3 shows representative spectra of proteins retained on the four array types prepared with SPA. When assigning peak clusters across the different array types, peaks on different surfaces were assumed to be the same protein if their respective *m/z *were within 0.3%. Nevertheless, it must be mentioned that without assigned peak identities one can never be confident that a peak of similar mass, observed between the different array types, represents the same protein. A Venn diagram representing peak counts, determined as unique or common across the array types, is shown in figure 4. A significant proportion of all detected peaks were found to be common to two or more array types. Approximately, 8 and 12 peaks were common to all array types processed with SPA and CHCA, respectively, out of which 6 peaks were common to all array types processed with both matrices (*m/z *8740, 11956, 12055, 13996, 14122 and 14164). Overall, approximately 75 and 56 unique peaks (defined as only present on one surface) were detected across all four arrays types prepared with SPA and CHCA, respectively. The highest number of unique peaks was observed on CM10 and IMAC30 arrays. Moreover, the combination of CM10 and IMAC30 covered 89% and 79% of all detected peaks on SPA and CHCA, respectively.

### Assessment of spectral reproducibility

**Figure 5:**
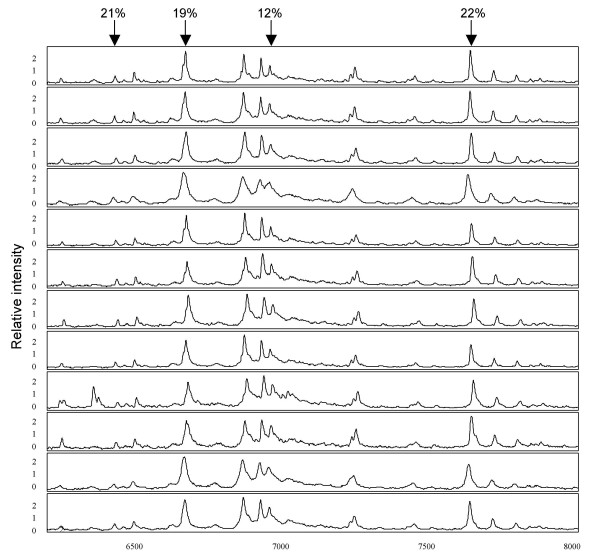
**Assessment of inter-chip variability. **Spectra were obtained from a CSF sample of equal volume loaded across twelve chips used for one bioprocessor plate, and CVs calculated on normalized peak intensities. Four peaks, representing various intensities, are indicated along with their calculated CVs across the 12 chips.

For comparative studies, reliable and reproducible protein profiles must be obtained to ensure that the variation in spectra reflects biological differences in protein concentration rather than systematic variability. As such, accurate mass peak heights are necessary, and the technical variation of the profiles must be known. In order to increase the reliability of the approach we adapted the entire processing of arrays to a robotics system for consistency. Intra-chip reproducibility was assessed using 6 technical replicates of a pooled CSF sample spotted in equal volumes (5μL) on four individual CM10 chips. A total of 30 randomly selected peaks with a signal-to-noise ratio >3, and common to all spectra, were randomly selected and compared with regards to their normalized peak intensities by calculating the CV within each chip. The mean CV for intra-chip variability was 17%, ranging from 7–34% across individual peaks. To evaluate the inter-chip variability, pooled CSF sample was randomly placed on a single spot across each of twelve different CM10 chips on one bioprocessor plate. This was repeated using a second bioprocessor plate processed the following day. A total of 37 peaks with a signal-to-noise ratio >3, and common to all spectra, were randomly selected and compared with regards to their normalized peak intensities by calculating the CV within each bioprocessor. Mean peak CVs of 19% (ranging from 6–29%) and 23% (ranging from 7–47%) for normalized intensity were calculated for each bioprocessor. The spectra profiles for the inter-chip assay are shown in figure 5. Overall, the CVs obtained for both inter- and intra-chip indicate that the processing of protein chips across a bioprocessor using a robotic system is reliable and reproducible.

**Figure 6:**
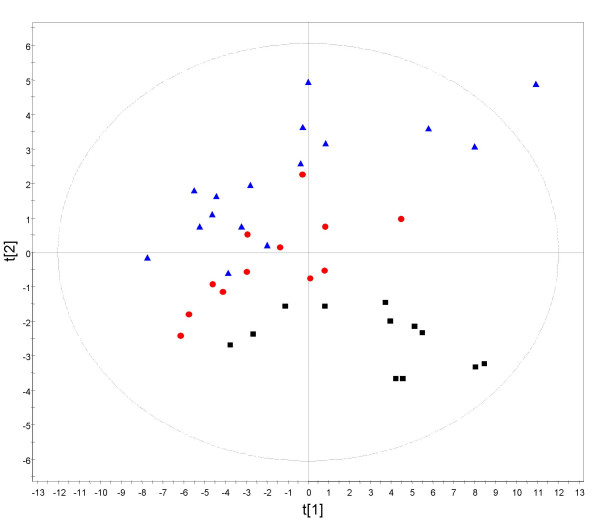
**Assessment of systematic variability across bioprocessor plates using principle component analysis. **Variability was evaluated across three bioprocessor plates processed 4 and 24 hours apart. Spectra were obtained from pooled CSF sample randomly placed on a single spot across each of twelve different CM10 chips on one bioprocessor plate. The same was repeated for the other plates 4 and 24 hr later. Following baseline subtraction, normalization and spectra alignment, 45 peaks which appeared in all spectra were used for PCA analysis. The PCA results were color coded for the three bioprocessor plates: blue, 0 hr; red, 4 hr; black, 24 h.

In addition we evaluated the variability across three bioprocessor plates processed 4 and 24 hours apart. Pooled CSF sample was randomly placed on a single spot across each of twelve different CM10 chips on one bioprocessor plate. The same was repeated for the other plates 4 and 24 h later. Figure 6 shows the principle component analysis results for data points representing spectra of each sample with color denoting the bioprocessor plate. The clustering of points to their respective plates indicates the presence of a discernable systematic variability across plates, with the largest separation between clusters seen for the plate processed at 24 h. This variability is very important in the context of large studies in which a large number of bioprocessor plates would be needed. Intelligent randomization procedures using technical replicates would need to be adopted in order to minimize any systematic bias introduced by plates and time of process.

## Discussion

Surface-based enrichment approaches in combination with MS, such as ProteinChip SELDI-TOF approach, have been developed to facilitate the high-throughput analysis of peptides and proteins in complex biological samples such as body fluids. ProteinChip SELDI-TOF technology allows for facile sample analysis since very small sample volumes can be directly applied to the ProteinChip array surfaces, and the process can be easily automated for high-throughput analysis. In the present study, we applied the ProteinChip SELDI-TOF approach coupled with an automated robotic sample preparation workstation as a strategy for potentially screening large numbers of CSF samples from clinical studies. In particular, we take advantage of the technologies high-throughput potential for screening proteins between the 2.5–20 kDa mass window range.

To date, few examples exist in the literature describing the application of ProteinChip SELDI-TOF approach for analyzing CSF [28-33]. and none describe a comparative evaluation of CSF profiles across different conditions and arrays. To our knowledge this paper is the first to describe a comparative investigation of experimental procedures for identifying effective and consistent conditions for retention of CSF proteins on different ProteinChip array types between the 2.5–20 kDa mass range. ProteinChip array types, along with sample and binding buffer conditions, and matrices were evaluated based on the number of resolved peaks exhibiting a signal-to-noise ratio of 3 or greater. We found that CSF prepared under denaturing conditions without reduction performed best on all ProteinChip arrays processed with either SPA or CHCA as the matrix. With respect to the selection of binding buffer for protein retention, buffer in the absence of Triton X-100 performed better on CM10 and Q10 arrays. For IMAC30 and H50 slightly higher peak numbers were obtained by surface activation with copper, and using 10% AcN/0.1% TFA as binding buffer, respectively. However, the type of matrix rather than the binding condition used on each chip surface appears to dictate the overall protein profile from each chip type. ProteinChip arrays prepared with SPA consistently showed better peak sharpness and higher peak number across all surfaces. The reliability of the approach was emphasized with the low CVs which compare favorably to the CVs reported for other protein profiling approaches [34]. It's very likely that a major contributor to this was the adaptation of the entire process of ProteinChip preparation to a robotics system. Nevertheless, we observed systematic variability across plates processed at different times. Therefore, to ensure that systematic bias is minimized, sample randomization procedures using technical replicates must be properly addressed when large studies are conducted

The surface-based enrichment approach using ProteinChips is a rapid and straightforward tool for screening peptides and small proteins below 20 kDa from small sample volumes. However, the major shortcomings of this approach are peak identities and limited proteome coverage. In this study, only a small subset of species, out of potentially 100s' if not 1000s' of circulating molecules in CSF, is actually monitored as peaks when the starting material is unfractionated. Moreover, due to the high level of overlap in profiles between the tested array types, we observed that the combination of CM10 and IMAC30 was sufficient enough to represent between 80–90% of all assigned peaks on the tested arrays. Preferably, we would have wanted a much lower overlap in profiles between the array types so as to increase proteome coverage.

CSF contains a tremendous array of molecules, spanning a concentration range of 10 orders of magnitude between the highest and lowest abundance proteins, of which only a handful (e.g. albumin) constitute up to 90% of the total protein concentration. Consequently, it is likely that the low abundance molecules of diagnostic potential will be competed out by high abundance non-informative molecules for binding on the solid-phase. Indeed, most differences identified by this approach in body fluids have shown bias towards the high abundant molecules (present in the μg/mL to mg/mL concentration range) implying that the ProteinChip SELDI-TOF technology is probably not adequate for 'deep' proteome analysis [19]. Initial prefractionation of CSF by LC based methods, in combination with immunodepletion of abundant proteins, are thus likely obligatory steps for exploiting low-abundant molecules of diagnostic potential [35,36]. Concurrently, a balance must be found between analysis depth, speed, throughput, and sample requirements.

Further developments in analytical strategies for selective protein absorption on solid support coupled to high mass accuracy and high resolution MS technology is necessary before this approach can be used as a more comprehensive proteomic profiling tool in an automated and high throughput fashion. A promising area of development is in the utilization of combinatorial ligands for mining the proteome [38,39]. Libraries of potential millions of discrete amino acid ligands synthesized on solid-phase beads have been created, in which theoretically, there is a ligand for every protein, antibody, and peptide present in the starting material. It is envisaged that these beads impregnated with complex proteomes could capture equal quantities of each and all the peptides and proteins present in CSF, thus reducing the concentration difference. This could make proteomic approaches using ProteinChips more adapted to 'deep' proteome analysis and biomarker discovery. Another interesting approach was adopted by both Mehta [39] and Zhou [40] to profile the proportion of low molecular weight species bound to specific circulating carrier proteins. It was found that by selectively targeting high abundant proteins in serum for depletion, many other peptides and small proteins associated with these abundant proteins are concomitantly removed. By this targeted selection, the concentrations of associated peptides and small proteins are enriched to levels of detection. Indeed, some of the species identified represented clinically relevant biomarkers, including prostate-specific antigen which in healthy males is present at a concentration of 1 ng/mL. Examination of the low molecular weight species bound to specific carrier proteins may, therefore, allow for the detection and mining of diagnostic information.

In spite of the current technical shortcomings of the ProteinChip SELDI-TOF technology, one could envisage the potential utilization of surface-based enrichment approaches in combination with MS as a strictly high throughput screening tool to drive decisions on sample selection prior to more in-depth discovery of diagnostic markers. For instance, the ProteinChip SELDI-TOF technology could be used as an upfront quality control step for screening large sample numbers obtained from multiple clinical centres. Multivariate analysis of the data sets would help reveal potential sample outliers as a result of either sample handling or intrinsic patient variability. This would aid in the selection of a smaller sample subset for more in-depth comparative analysis using alternative proteomic platforms such as multidimensional LC-MS based strategies.

## Conclusion

In conclusion, we have shown that the ProteinChip SELDI-TOF technology can provide a fast, robust, straightforward and reproducible profiling platform for measuring peaks in the low molecular mass range of the CSF proteome. We are currently examining the robustness of the profile across patient sample sets from healthy, mild cognitive impaired, AD, and other dementias in order to address the feasibility of the current platform in combination with decision algorithms to detect biomarker panels associated with the different pathological conditions, and as a screening tool for sample selection prior to more in-depth analysis.

## Materials and methods

### Cerebrospinal fluid samples

Normal CSF samples obtained from consenting patients were provided by PrecisionMed Inc. (San Diego, CA). CSF samples were obtained by lumbar puncture as part of a routine clinical procedure. The samples were collected in polypropylene tubes and gently mixed to avoid gradient effects. The samples were centrifuged at 2000 × g for 10 min to remove cells and other insoluble material. Supernatants were frozen in aliquots and stored at -80°C until analysis.

### Sample preparation

A standard pooled Human CSF sample was used to evaluate the different conditions of retention on the all ProteinChip arrays. CSF samples were prepared on four different ProteinChip array surfaces: cation-exchange (CM10), strong anion-exchange (Q10); metal-binding (IMAC30) and reverse phase (H50). All ProteinChip Arrays were processed on the same day following the procedures recommended by the manufacturer. Binding buffers used for the different arrays were 100 mM ammonium acetate pH 4.0 (with or without 0.1% Triton X-100) for CM10; 100 mM Tris-HCl pH 9.0 (with or without 0.1% Triton X-100) for Q10; 100 mM Na phosphate, 500 mM NaCl pH 7.0 (activated with either 100 mM copper sulphate or 100 mM nickel sulphate hexahydrate) for IMAC30., 10% acetonitrile (AcN), 0.1% trifluoroacetic acid (TFA) or phosphate buffered saline (PBS) for H50.

Preliminary experiments were first performed on ProteinChip arrays spotted with different CSF sample volumes prepared in different ratios of sample to binding buffer. Based on the overall number of detected peaks and their associated intensities we determined 5 μL of CSF diluted 1:4 in the appropriate binding buffer to be the optimal amount for loading onto the ProteinChip arrays (data not shown). Subsequent experiments reported in this paper were performed using this CSF volume. In brief, 5 μL of CSF was diluted 1:1 in sample buffer preparations representing the following conditions: native (dH_2_O), denatured (9.5 M urea, 2% CHAPS, 50 mM Tris-HCl, pH 9.0) and denatured/reduced (9.5 M urea, 2% CHAPS, 50 mM Tris-HCl, 10 mM dithiothreitol, pH 9.0). Following 20 min incubation at 4°C, the CSF samples were then added to separate spots on the array surface using a Biomek laboratory station (Beckman-Coulter, CA) modified to make use of a ProteinChip array bioprocessor (Ciphergen Biosystems Inc.). The samples on the arrays were diluted 1:4 in the appropriate ProteinChip array binding buffer. The bioprocessor was then centrifuged for 10 s at 1000 rpm, using an Eppendorf Centrifuge 5804 system, to remove any air bubbles. The arrays were incubated for 1 h at room temperature with gentle shaking. The ProteinChip arrays were washed twice with 50 μL binding buffer for 5 minutes with gentle shaking, followed by two washes with 150 μL distilled water for 1 minute to remove buffer salts. The bioprocessor was subsequently removed and the ProteinChip arrays air-dried at 23°C for 15 minutes. Once dry, two 1 μL aliquots of a 50% saturated sinapinic acid (SPA; Ciphergen Biosystems Inc.) solution prepared in 50% acetonitrile and 0.5% TFA was added to each spot of the ProteinChip array. The arrays were allowed to air-dry before SELDI analysis. The same was repeated for ProteinChip arrays prepared with 50% saturated α-Cyano-4-hydroxycinnamic acid (CHCA; Ciphergen Biosystems Inc.) solution prepared in 50% acetonitrile and 0.5% TFA. Each condition was analysed in triplicate. For storage, the spotted arrays were kept in the dark at room temperature.

### Data acquisition and spectral processing

ProteinChip arrays were placed in the ProteinChip reader Series 4000 mass spectrometer (Ciphergen Biosystems Inc.) and mass spectra was acquired using settings optimized for the *m/z *range of 2.5–20 kDa. For each spot around 175 shots were collected in positive ionization mode using a laser intensity set at 1,500 nJ. For SPA preparations a deflector setting of 1000 Da, and an ion focus mass of 9000 Da was used, whereas for CHCA preparations a deflector setting of 500 Da, and an ion focus mass of 3500 Da was used. The spectra were externally calibrated using the "All-In-One" peptide mass standard (Ciphergen Biosystems Inc.). The standards, ranging from 1–7 kDa, were prepared on NP20 ProteinChip arrays according to the manufactures recommendation. The ProteinChip reader was calibrated daily and we typically achieved mass accuracies within 150 ppm.

Spectra were analysed using Ciphergen Express software Version 3.0.5 (Ciphergen Biosystems Inc.). The baseline was subtracted (baseline smooth width of 25) and the spectral intensities were normalized by total ion current (TIC) to an external normalization coefficient of 0.2 between the mass range of 2.5 to 20 kDa. Automatic peak detection was performed using the following settings: noise calculation between the mass range of 2.5 to 20 kDa, 3 times the signal-to-noise ratio and 2 times the valley depth for the first pass, and 2 times the signal-to-noise ratio and 2 times the valley depth for the second pass. In addition to the automatic assignment of peaks, manual inspection of the spectra was conducted as a quality control step to ensure that all peaks were correctly labelled. When assigning peak clusters across spectra, two peaks on different surfaces were assumed to be the same protein if both their respective *m/z *were within 0.3%. Principle component analysis was performed using the SIMCA-P statistical package (Umetrics AB, Sweden), and was used to reveal major variance structure and clustering.

## Abbreviations

SELDI-TOF-MS, Surface Enhanced Laser Desorption/Ionization-Time-of-flight-Mass Spectrometry; CSF, cerebrospinal fluid; CNS, central nervous system; CM10, weak cation exchange; Q10, strong anion exchange; IMAC30, immobilized metal affinity capture; H50, reverse phase; SPA, sinapinic acid ; CHCA, α-Cyano-4-hydroxycinnamic acid; *m/z*, mass-to-charge ratio; DTT, dithiothreitol.

## Authors' contributions

NG participated in the design of the study and drafted the manuscript. SC carried out all the SELDI-TOF experiments. BGM participated in the design of the study and helped to draft the manuscript.

## References

[B1] Zheng W, Chodobski A (2005). The Blood-Cerebrospinal Fluid Barrier.

[B2] Thompson EJ (2004). Cerebrospinal Fluid: CSF Proteins and Their Relevance in Research, Diagnostics and Treatment.

[B3] Davidsson P, Sjogren M (2005). The use of proteomics in biomarker discovery in neurodegenerative diseases. Dis Markers.

[B4] Rohlff C (2001). Proteomics in neuropsychiatric disorders. Int J Neuropsychopharmacol.

[B5] Romeo MJ, Espina V, Lowenthal M, Espina BH, Petricoin EFIII, Liotta LA (2005). CSF proteome: a protein repository for potential biomarker identification. Expert Rev Proteomics.

[B6] Siman R, McIntosh TK, Soltesz KM, Chen Z, Neumar RW, Roberts VL (2004). Proteins released from degenerating neurons are surrogate markers for acute brain damage. Neurobiol Dis.

[B7] Lamerz J, Selle H, Scapozza L, Crameri R, Schulz-Knappe P, Mohring T, Kellmann M, Khamenia V, Zucht HD (2005). Correlation-associated peptide networks of human cerebrospinal fluid. Proteomics.

[B8] Ramstrom M, Ivonin I, Johansson A, Askmark H, Markides KE, Zubarev R, Hakansson P, Aquilonius SM, Bergquist J (2004). Cerebrospinal fluid protein patterns in neurodegenerative disease revealed by liquid chromatography-Fourier transform ion cyclotron resonance mass spectrometry. Proteomics.

[B9] Wenner BR, Lovell MA, Lynn BC (2004). Proteomic analysis of human ventricular cerebrospinal fluid from neurologically normal, elderly subjects using two-dimensional LC-MS/MS. J Proteome Res.

[B10] Yuan X, Desiderio DM (2005). Proteomics analysis of human cerebrospinal fluid. J Chromatogr B Analyt Technol Biomed Life Sci.

[B11] Zhang J, Goodlett DR, Quinn JF, Peskind E, Kaye JA, Zhou Y, Pan C, Yi E, Eng J, Wang Q, Aebersold RH, Montine TJ (2005). Quantitative proteomics of cerebrospinal fluid from patients with Alzheimer disease. J Alzheimers Dis.

[B12] Grus FH, Podust VN, Bruns K, Lackner K, Fu S, Dalmasso EA, Wirthlin A, Pfeiffer N (2005). SELDI-TOF-MS ProteinChip array profiling of tears from patients with dry eye. Invest Ophthalmol Vis Sci.

[B13] Kozak KR, Amneus MW, Pusey SM, Su F, Luong MN, Luong SA, Reddy ST, Farias-Eisner R (2003). Identification of biomarkers for ovarian cancer using strong anion-exchange ProteinChips: potential use in diagnosis and prognosis. Proc Natl Acad Sci U S A.

[B14] Pawlik TM, Fritsche H, Coombes KR, Xiao L, Krishnamurthy S, Hunt KK, Pusztai L, Chen JN, Clarke CH, Arun B, Hung MC, Kuerer HM (2005). Significant differences in nipple aspirate fluid protein expression between healthy women and those with breast cancer demonstrated by time-of-flight mass spectrometry. Breast Cancer Res Treat.

[B15] Petricoin EF, Liotta LA (2004). SELDI-TOF-based serum proteomic pattern diagnostics for early detection of cancer. Curr Opin Biotechnol.

[B16] Sauter ER, Shan S, Hewett JE, Speckman P, Du Bois GC (2005). Proteomic analysis of nipple aspirate fluid using SELDI-TOF-MS. Int J Cancer.

[B17] Schaub S, Rush D, Wilkins J, Gibson IW, Weiler T, Sangster K, Nicolle L, Karpinski M, Jeffery J, Nickerson P (2004). Proteomic-based detection of urine proteins associated with acute renal allograft rejection. J Am Soc Nephrol.

[B18] Merchant M, Weinberger SR (2000). Recent advancements in surface-enhanced laser desorption/ionization-time of flight-mass spectrometry. Electrophoresis.

[B19] Diamandis EP, van der Merwe DE (2005). Plasma protein profiling by mass spectrometry for cancer diagnosis: opportunities and limitations. Clin Cancer Res.

[B20] Issaq HJ, Veenstra TD, Conrads TP, Felschow D (2002). The SELDI-TOF MS approach to proteomics: protein profiling and biomarker identification. Biochem Biophys Res Commun.

[B21] Pusch W, Flocco MT, Leung SM, Thiele H, Kostrzewa M (2003). spectrometry-based clinical proteomics. Pharmacogenomics.

[B22] Brouwers FM, Petricoin EFIII, Ksinantova L, Breza J, Rajapakse V, Ross S, Johann D, Mannelli M, Shulkin BL, Kvetnansky R, Eisenhofer G, Walther MM, Hitt BA, Conrads TP, Veenstra TD, Mannion DP, Wall MR, Wolfe GM, Fusaro VA, Liotta LA, Pacak K (2005). Low molecular weight proteomic information distinguishes metastatic from benign pheochromocytoma. Endocr Relat Cancer.

[B23] Rai AJ, Zhang Z, Rosenzweig J, Shih I, Pham T, Fung ET, Sokoll LJ, Chan DW (2002). Proteomic approaches to tumor marker discovery. Arch Pathol Lab Med.

[B24] Schaub S, Wilkins JA, Antonovici M, Krokhin O, Weiler T, Rush D, Nickerson P (2005). Proteomic-based identification of cleaved urinary beta2-microglobulin as a potential marker for acute tubular injury in renal allografts. Am J Transplant.

[B25] Wulfkuhle JD, Liotta LA, Petricoin EF (2003). Proteomic applications for the early detection of cancer. Nat Rev Cancer.

[B26] Tirumalai RS, Chan KC, Prieto DA, Issaq HJ, Conrads TP, Veenstra TD (2003). Characterization of the low molecular weight human serum proteome. Mol Cell Proteomics.

[B27] Villanueva J, Philip J, Entenberg D, Chaparro CA, Tanwar MK, Holland EC, Tempst P (2004). Serum peptide profiling by magnetic particle-assisted, automated sample processing and MALDI-TOF mass spectrometry. Anal Chem.

[B28] Carrette O, Demalte I, Scherl A, Yalkinoglu O, Corthals G, Burkhard P, Hochstrasser DF, Sanchez JC (2003). A panel of cerebrospinal fluid potential biomarkers for the diagnosis of Alzheimer's disease. Proteomics.

[B29] Gineste C, Ho L, Pompl P, Bianchi M, Pasinetti GM (2003). High-throughput proteomics and protein biomarker discovery in an experimental model of inflammatory hyperalgesia: effects of nimesulide. Drugs.

[B30] Sanchez JC, Guillaume E, Lescuyer P, Allard L, Carrette O, Scherl A, Burgess J, Corthals GL, Burkhard PR, Hochstrasser DF (2004). Cystatin C as a potential cerebrospinal fluid marker for the diagnosis of Creutzfeldt-Jakob disease. Proteomics.

[B31] Mannes AJ, Martin BM, Yang HT, Keller JM, Lewin S, Gaiser RR, Iadarola MJ (2003). Cystatin C as a cerebrospinal fluid biomarker for pain in humans. Pain.

[B32] Ranganathan S, Williams E, Ganchev P, Gopalakrishnan V, Lacomis D, Urbinelli L, Newhall K, Cudkowicz ME, Brown RH, Bowser R (2005). Proteomic profiling of cerebrospinal fluid identifies biomarkers for amyotrophic lateral sclerosis. Journal of Neurochemistry.

[B33] Ruetschi U, Zetterberg H, Podust VN, Gottfries J, Li S, Simonsen AH, McGuire J, Karlsson M, Rymo L, Davies H, Minthon L, Blennow K (2005). Identification of CSF biomarkers for frontotemporal dementia using SELDI-TOF. Experimental Neurology.

[B34] Hansson SF, Puchades M, Blennow K, Sjogren M, Davidsson P (2004). Validation of a prefractionation method followed by two-dimensional electrophoresis – Applied to cerebrospinal fluid proteins from frontotemporal dementia patients. Proteome Sci.

[B35] Maccarrone G, Milfay D, Birg I, Rosenhagen M, Holsboer F, Grimm R, Bailey J, Zolotarjova N, Turck CW (2004). Mining the human cerebrospinal fluid proteome by immunodepletion and shotgun mass spectrometry. Electrophoresis.

[B36] Maccarrone G, Birg I, Malisch E, Rosenhagen MC, Ditzen C, Chakel JA, Mandel F, Reimann A, Doertdudak CC, Haegler K, Holsboer F, Turck CW (2004). In-depth analysis of the human CSF proteome using protein prefractionation. Clinical Proteomics Journal.

[B37] Righetti PG, Castagna A, Antonucci F, Piubelli C, Cecconi D, Campostrini N, Rustichelli C, Antonioli P, Zanusso G, Monaco S, Lomas LBoschetti E (2005). Proteome analysis in the clinical chemistry laboratory: myth or reality?. Clin Chim Acta.

[B38] Righetti PG, Castagna A, Antonioli P, Boschetti E (2005). Prefractionation techniques in proteome analysis: the mining tools of the third millennium. Electrophoresis.

[B39] Mehta AI, Ross S, Lowenthal MS, Fusaro V, Fishman DA, Petricoin EFIII, Liotta LA (2003). Biomarker amplification by serum carrier protein binding. Dis Markers.

[B40] Zhou M, Lucas DA, Chan KC, Issaq HJ, Petricoin EF, Liotta LA, Veenstra TD, Conrads TP (2004). An investigation into the human serum "interactome". Electrophoresis.

